# Gene co-expression network and differential expression analyses reveal key genes for weaning weight in Simmental-Holstein crossbred cattle

**DOI:** 10.1080/10495398.2024.2404042

**Published:** 2024-09-20

**Authors:** Saina Yan, Fen Pei, Jingfnag Si, Md. Yousuf Ali Khan, Sihai Ou, Yang Yang, Zongsheng Zhao, Alfredo Pauciullo, Yi Zhang

**Affiliations:** a College of Animal Science and Technology, China Agricultural University, Beijing, China; b Animal Husbandry and Veterinary Station of the 8th Division, Shihezi City, Xinjiang Production and Construction Corps, Shihezi, China; c College of Animal Science and Technology, Shihezi University, Shihezi, China; d Department of Agricultural, Forest and Food Sciences, University of Torino, Grugliasco, TO, Italy

**Keywords:** RNA-seq, Simmental-Holstein crossbred cattle, weaning weight, WGCNA

## Abstract

Weaning weight is a key indicator of the early growth performance of cattle. An understanding of the genetic mechanisms underlying weaning weight will help increase the accuracy of selection of breeding animals. In order to identify candidate genes associated with weaning weight in Simmental-Holstein crossbred cattle, this study generated RNA-Sequencing (RNA-seq) data from 86 crossbred calves (37 males and 49 famales) and measured their weaning weight and body size traits (wither height, body length, chest girth, rump width, and rump length). Differential gene expression analysis and weighted gene co-expression network analysis (WGCNA) were performed. A total of 498 differentially expressed genes (DEGs) were identified between the low weaning weight (LWW) group and the high weaning weight (HWW) group. Weaning weight was transcriptionally correlated (FDR < 0.05) with four of the eleven co-expression gene modules. By intersecting DEGs and hub genes of the four modules, we identified a final set of 37 candidate genes enriched in growth, development, or immune-related processes. In addition, one co-expression module was significantly correlated with all the five body size traits (P < 0.05), from which *MX1* was identified as a key candidate gene through protein-protein interaction (PPI) analysis of hub genes. Further evidence from cattle transcriptome-wide association study analysis (TWAS) and human phenome-wide association study (PheWAS) validated significant associations of *CACNA1S*, *SEMA7A*, *VCAN*, *CD101*, *CD19*, and *CSF2RB* with growth and development traits (P < 0.05). Notably, *CACNA1S* and *CD19* were also associated with typical immune traits such as B cell proliferation, differentiation, and activation. In conclusion, this study reveals new candidate genes significantly associated with weaning weight in Simmental-Holstein crossbred cattle, providing a basis for further exploration of the genetic mechanisms behind growth traits of cattle.

## Introduction

Holstein is a dominant dairy cattle breed worldwide, with high milk productivity.^1^ Simmental is a dual-purpose breed, valued for both milk and meat and characterized by high milk components, longevity, strong fertility, and adaptive ability.^2^ Crossbreeding is an effective way to enhance the performance of dairy cattle.^3^ Previous studies have shown that the crossbred cattle of Simmental and Holstein (S × H) exhibited improved milk composition, udder health, reproductive capability, body condition scoring, and longevity.^1^^,^^3–9^ In addition, crossbreeding is a step forward to create a new composite cattle breed with enhanced traits.^10^^,^^11^ Weaning weight is a key indicator of early calf growth traits, impacting heifer maturity, reproduction, milk production, and health traits.^12^^,^^13^ As an economically important quantitative trait, weaning weight is controlled by polygenes and complex genetic regulatory mechanisms.^14^ However, the genetic mechanisms and functional genes underlying weaning weight are poorly understood in cattle.

Transcriptomic sequencing (RNA sequencing, RNA-seq) has long been considered an effective way to discover functional genes.^15^ Weighted Gene Co-expression Network Analysis (WGCNA) is a widely used bioinformatics method.^16^ Based on high-throughput RNA expression data, this method initially weights genes according to their relative expression levels across multiple samples to form weighted connections, from which a co-expression network is constructed to identify sub-networks (or gene modules). When the gene modules are tested to be significantly correlated with phenotype, the hub genes in the modules are considered as important functional genes.^17–19^

**Figure 1.**
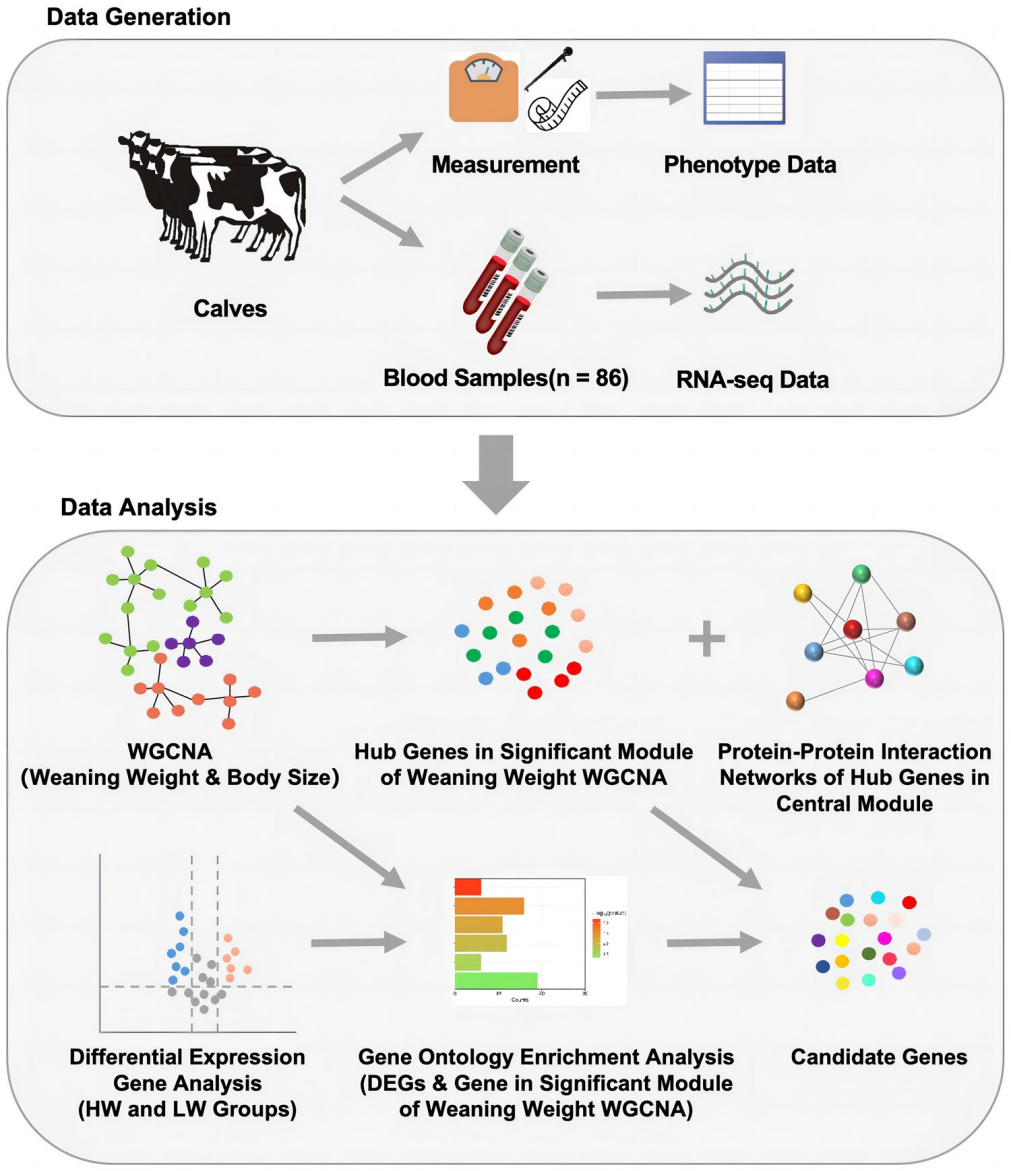
Workflow of the study.

Weaning is a necessary management practice for calf. Using transcriptomic analysis, O’Loughlin et al. reported genes associated with the stress response to weaning.^20^ However, to our best knowledge, no transcriptomic studies were reported on the weaning weight of S × H cattle. As blood is a tissue closely related to growth and immunity^21^^,^^22^, and blood samples could be conveniently collected *in vivo*, this study, based on RNA-seq, aimed to identify significant candidate genes for weaning weight in S × H cattle and explore the genetic mechanisms underlying weaning weight (Fig. 1).

## Materials and methods

### Animals and phenotypic data

The study population consisted of 86 calves (37 males and 49 females) generated by crossbreeding of German Simmental (Fleckvieh) with Holstein (S × H) in Xinjiang, China. These calves were raised in the same farm. The weaning weight was recorded on the day of weaning at the age of approximately two months, along with body size measurements (body length, wither height, chest girth, rump length, and rump width) (Fig. 1, Table S1). Out of 86 individuals, 78 had data on both weaning weight and body size, while the remaining 8 lacked body size measurement data (Table 1, Table S1).

**Table 1.** Descriptive statistics for weaning weight and body sizes of S × H cattle.

Traits	n	Mean	SD
Actual weaning weight (kg)	86	90.51	10.73
Body length (cm)	78	91.54	3.71
Wither height (cm)	78	91.86	5.28
Chest girth (cm)	78	103.32	5.06
Rump length (cm)	78	28.96	1.64
Rump width (cm)	78	21.56	1.35

To account for the effects of birth weight, weaning age, weaning season, and sex on weaning weight, a linear regression model was constructed using the *lm* function in R v4.3.2. The model was as follows:

$$y_{\text{ijklm}}=\beta_{0}+\beta_{1}x_{i}+\beta_{2}x_{j}+x_{k}+x_{l}+e_{\text{ijklm}},$$

where, $y_{\text{ijklm}}$ is the vector of observed weaning weights; $x_{i}$ is the vector of birth weights; $x_{j}$ is the vector of the weaning ages; $x_{k}$ is the vector of genders (male, female); $x_{l}$ is the vector of weaning seasons, namely spring (March to May), summer (June to August), and autumn (September to October); $\beta_{0}$ is the intercept, $\beta_{1}$ and $\beta_{2}$ are regression coefficients, and $e_{\text{ijklm}}$ is the residual.

### Blood samples collection and RNA-seq

Blood samples were collected from the jugular vein on the day of weaning and stored in 2.5 mL PAXgene Blood RNA tubes (PreAnalytiX GmbH, Switzerland) at −18 °C. Total RNA was extracted from the blood using Trizol (Invitrogen, USA), followed by purification by the AMPure XP system (Backman Coulter, USA). Concentration and integrity of total RNA were evaluated using NanoDrop 2000 spectrophotometer (Thermo Fisher Scientific, USA) and Agilent Bioanalyzer (Agilent Technologies, USA), respectively. Pair-end libraries were constructed and sequenced on the Illumina NovaSeq 6000 platform to generate paired-end reads of 2 × 150 bp (Beijing Glbizzia Biotechnology Co. Ltd., China).

### RNA-seq data processing

Quality control and filtering were performed using Trimmomatic v0.39^23^ with the parameters ‘adapters/TruSeq3-SE.fa:2:30:10 LEADING:3 TRAILING:3 SLIDINGWINDOW:4:15 MINLEN:36’ and resulted in 1,842,848,208 clean reads. The filtered data were then aligned to the ARS.UCD.1.2 bovine reference genome using STAR v2.7.9a,^24^ with an average mapping rate of 92.07% (Table S2). Quantification of the reads was performed by StringTie v2.1.5,^25^ which generated an expression matrix of 27,607 genes. The data were normalized to screen out genes with a threshold of Transcripts Per Million (TPM) > 1, in the top 75% of the median absolute deviation (MAD) and MAD greater than 0.01. A total of 12,524 and 12,419 genes were retained for WGCNA analyses with weaning weight of 86 animals and body size measurement of 78 animals, respectively.

### DEG analysis

The DESeq2 v1.42.0 package^26^ was used to analyze the differentially expressed genes (DEGs) between the low weaning weight (LWW) group and the high weaning weight (HWW) group. The weaning weights were sorted from lowest to highest, with the bottom 20 and the top 20 individuals designated as LWW and HWW, respectively (Table S3). The DEGs were filtered based on cutoff values: |log_2_FoldChange| ≥ 1 and adjusted p-value (p_adj_) ≤ 0.05. The ggplot2 R package was used to visualize the results as a volcano plot.

### WGCNA and gene ontology (GO) enrichment analysis

The analysis was conducted using the WGCNA package.^27^ First, an appropriate soft-thresholding power was identified using the pickSoftThreshold function with the option of scale-free fit index SFT.R.sq > 0.85 and average connectivity mean.k < 100. Then, a network was constructed using the blockwiseModules function with parameters set for minModuleSize of 30 and mergeCutHeight of 0.25 to cluster genes into co-expression modules. Module Eigengenes (MEs) were computed using the moduleEigengenes function and their correlation with phenotype was assessed. Finally, the hub genes in each module were selected based on the parameters: gene significance |GS| > 0.2 and module membership |MM| > 0.8.

The bovine gene IDs were mapped to their corresponding ENSEMBL human database IDs (org.Hs.eg.db). The enrichGO function of the clusterProfiler package^28^ in R was then used for gene ontology (GO) enrichment analysis to explore the main biological functions of the genes.

### Candidate genes identification

Candidate genes for weaning weight in S × H cattle were identified by selecting the overlapped genes between the DEGs enriched in growth and development and immune-related pathways and the hub genes of significant modules correlated with weaning weight and enriched in growth and development and immune-related pathways (Fig. 1).

Since the five body size traits are highly correlated, the genes and modules are shared across these traits. To effectively identify the candidate genes for the multiple body measurement traits, a protein-protein interaction (PPI) analysis was performed on the hub genes of significant modules using the STRING online database (http://string-db.org). The highly connected genes in the PPI network were considered as the key candidate genes (Fig. 1).

## Results

### Identification of candidate genes for weaning weight based on DEG analysis and WGCNA

**Figure 2.**
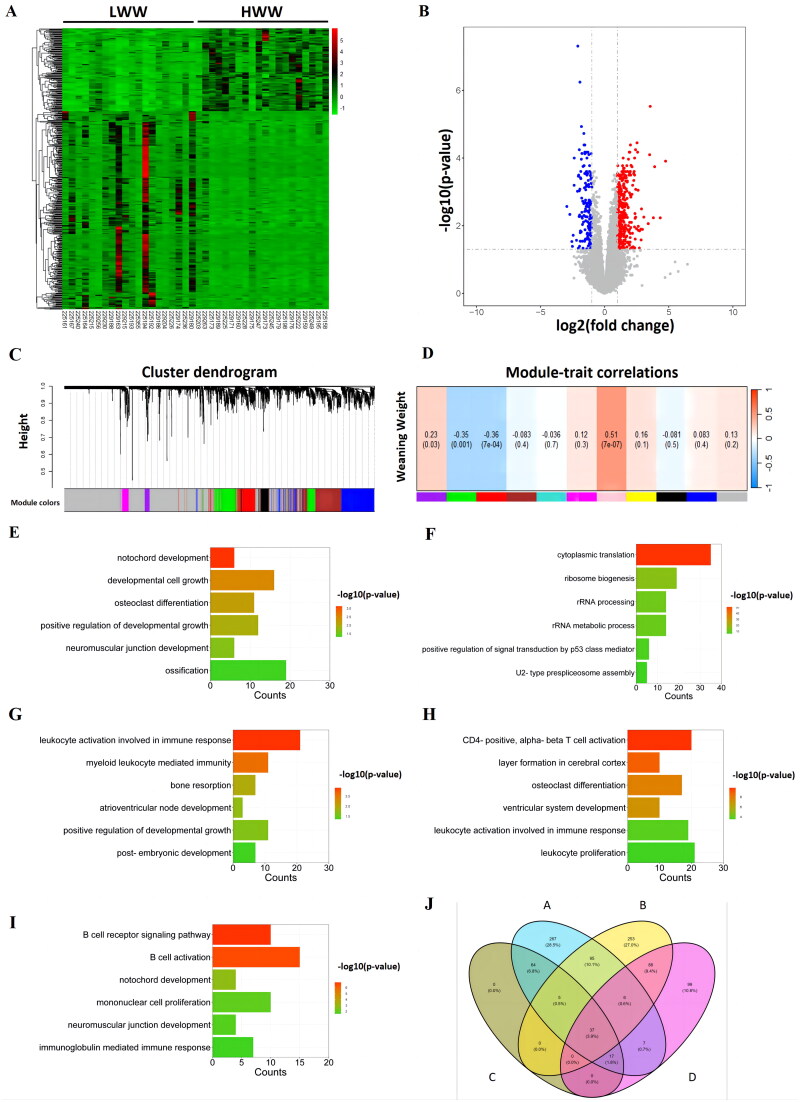
Identification of candidate genes for weaning weight based on differential expression analyses and WGCNA. (A) Heatmap of DEGs clustering for the high (HWW) and the low (LWW) weaning weight groups. (B) Volcano plot of DEGs for high and low weaning weight groups with blue dots representing downregulated genes and red dots representing upregulated genes. (C) Dendrogram of gene co-expression modules from 86 samples. (D) Correlations between weaning weight and gene co-expression modules. (E) Biological processes of growth and development the DEGs enriched in. (F–I) Biological processes of growth, development or immune response that the significant modules Purple, Green, Red or Pink enriched in, respectively. (J) Venn diagram of gene sets intersection. Subset A: genes of WGCNA modules that correlated with weaning weight and enriched in processes related to growth and development or immunity; Subset B: DEGs from high and low weaning weight groups; Subset C: Hub genes from significant weaning weight WGCNA modules; Subset D: DEGs from high and low weaning weight groups enriched in processes related to growth and development or immunity.

DEG analysis revealed differences in gene expression patterns between LWW and HWW (Fig. 2A). A total of 350 significantly upregulated genes and 148 downregulated genes were detected in LWW compared to HWW (Fig. 2B). WGCNA identified 11 gene co-expression modules (Fig. 2C). Correlation analysis between weaning weight and the MEs of these modules revealed four significant modules: Purple, Green, Red and Pink (Fig. 2D), of which 23, 224, 166, and 71 hub genes were identified, respectively.

GO analysis showed that the DEGs were significantly enriched in biological processes related to growth and development, including notochord development, developmental cell growth, osteoclast differentiation, positive regulation of developmental growth, neuromuscular junction development, and ossification (Fig. 2E). The genes of the four significant modules were significantly enriched in biological processes related to growth and development or immunity, such as osteoclast differentiation, bone resorption, notochord development, post-embryonic development, positive regulation of developmental growth, immunoglobulin mediated immune response, leukocyte activation involved in immune response, CD4 positive, alpha-beta T cells activation, myeloid leukocyte mediated immunity, and positive regulation of signaling by p53 class mediators (Fig. 2F–1).

By combining DEG analysis and WGCNA, a total of 130 genes were identified from the intersections of DEGs and hub genes of four significant modules. These genes were overlapped with genes enriched in growth and development or immune-related processes from DEGs and hub genes of four significant co-expression modules to result in 37 candidate genes (Fig. 2J), including *KAT2B*, *BCL6*, *ADGRG3*, *CSF2RB*, *SEMA7A*, *IRS2*, *ACVR1B*, *VCAN*, *TIAM1*, *CD101*, *VLDLR*, *CCR1*, *TXNIP*, *NIN*, *C5AR1*, *FOSL2*, *PTPRJ*, *PTAFR*, *C5AR2*, *CXCR2*, *PGLYRP4*, *CAPN3*, *MAPK13*, *ALOX5AP*, *DYSF*, *SLC11A1*, *CEBPB*, *CD79A*, *MS4A1*, *FCRL1*, *SERPING1*, *ERBB2*, *TNFRSF13C*, *ID3*, *CD19*, *CD79B*, *CACNA1S*.

### Identification of candidate genes for body size measurements based on WGCNA

**Figure 3.**
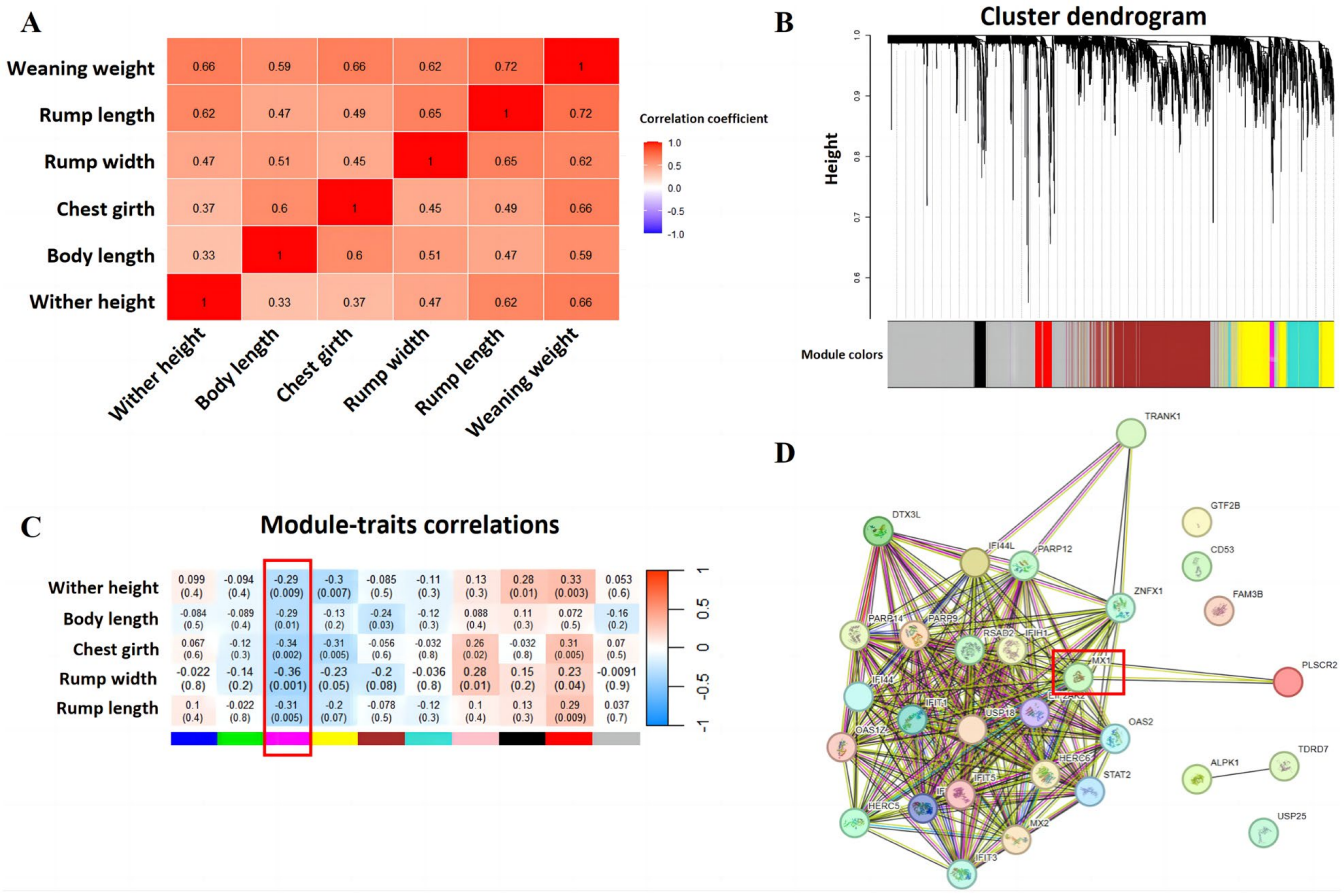
Identification of candidate genes for body size measurements. (A) Pairwise correlations between weaning weight and body size measurement traits in Simmental-Holstein crossbred cattle. (B) Dendrogram of gene co-expression modules from 78 samples. (C) Heatmap of correlations between body size measurements and gene co-expression modules. (D) PPI network diagram of hub genes from the Magenta module that were significantly correlated with body size measurement.

Given the strong positive correlation between body size measurements and weaning weight (Fig. 3A), we further explored gene co-expression modules correlated with body size measurements. The WGCNA of the 78 samples with body size measurements revealed 10 gene co-expression modules (Fig. 3B). Correlation analysis of these modules with phenotype identified four significant modules (Magenta, Yellow, Black, and Red) for wither height, two modules (Magenta and Brown) for body length, four modules (Magenta, Yellow, Pink, and Red) for chest girth, four modules (Magenta, Yellow, Pink, and Red) for rump width, and two modules (Magenta and Red) for rump length (Fig. 3C). The Magenta module, significantly correlated with all the five body measurements (P < 0.05), was considered as a key central module.

From the central module, a total of 27, 26, 31, 30, and 28 hub genes were identified for wither height, body length, chest girth, rump width, and rump length phenotypes, respectively. Finally, a PPI network was constructed using the identified hub genes (Fig. 3D). Based on the network connectivity (Table S4), the *MX1* was identified as a key candidate gene with an higher node degree.

### Cattle transcriptome-wide association study (TWAS) analysis and human phenome-wide association study (PheWAS) of candidate genes

**Figure 4.**
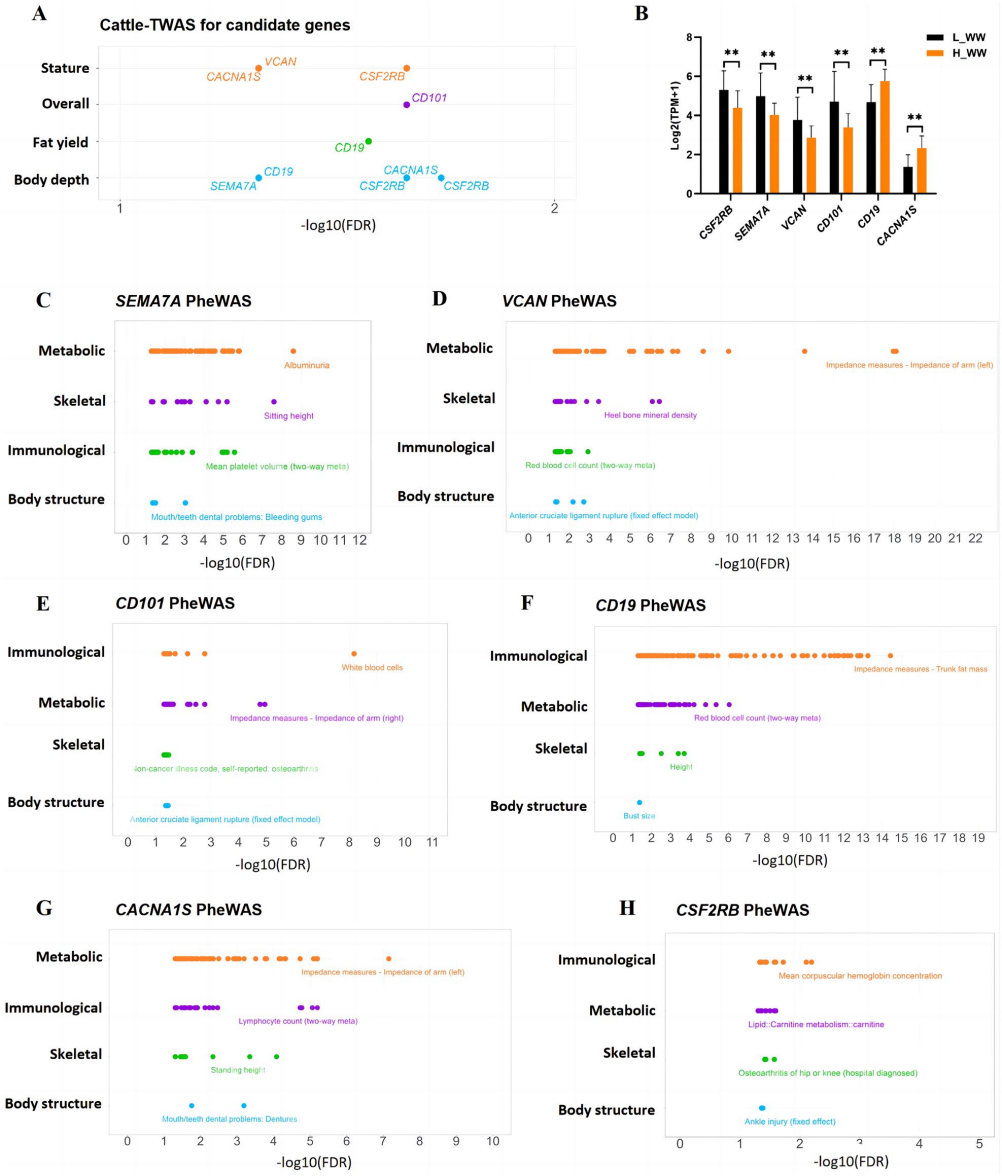
Transcriptome-wide association study (TWAS) analysis and phenome-wide association analysis (Phe-WAS) validated the candidate genes. (A) TWAS of genes associated with cattle growth and development. (B) Expression levels of genes *CSF2RB, SEMA7A, VCAN, CD101, CD19*, and *CACNA1S* in high (HWW) and low (LWW) weaning weight groups. (C–H) Phe-WAS results for genes *CACNA1S, SEMA7A, VCAN, CD101, CD19,* and *CSF2RB* in human, respectively.

Further analysis of the candidate genes using the cattle TWAS database (cattle GTEx, http://cgtex.roslin.ed.ac.uk/) revealed significant associations of the genes *SEMA7A, VCAN, CD101, CD19, CACNA1S* and *CSF2RB* with traits related to body depth, fat production, overall structure, and stature, which are relevant to cattle growth and development (Fig. 4A). Moreover, *CD19* and *CACNA1S* exhibited significantly high expression (P < 0.01) in HWW, while the other four genes exhibited significantly low expression (P < 0.01) (Fig. 4B).

To explore whether these six candidate genes possess similar functions in human, the PheWAS analysis was conducted on their human orthologs via the human PheWAS database (https://atlas.ctglab.nl/). The results showed that all the six genes were significantly associated with traits related to body structure, skeleton, immunology, and metabolism, which were closely linked to growth and development (Fig. 4C–H). Specifically, *SEMA7A*, *CD19* and *CACNA1S* were significantly associated with sitting height, height, and standing height, with *P*-values of 3.18E-10, 6.40E-05, and 9.34E-06, respectively; *VCAN* was significantly associated with heel bone mineral density (*P* = 2.34E-08); *CD19* was also significantly related to bust size (*P* = 0.04); *CD101* and *CACNA1S* were significantly associated with white blood cells and lymphocyte count, with *P*-values of 4.72E-11 and 1.70E-07, respectively.

### Correlation analysis between candidate genes expression and weaning weight

**Figure 5.**
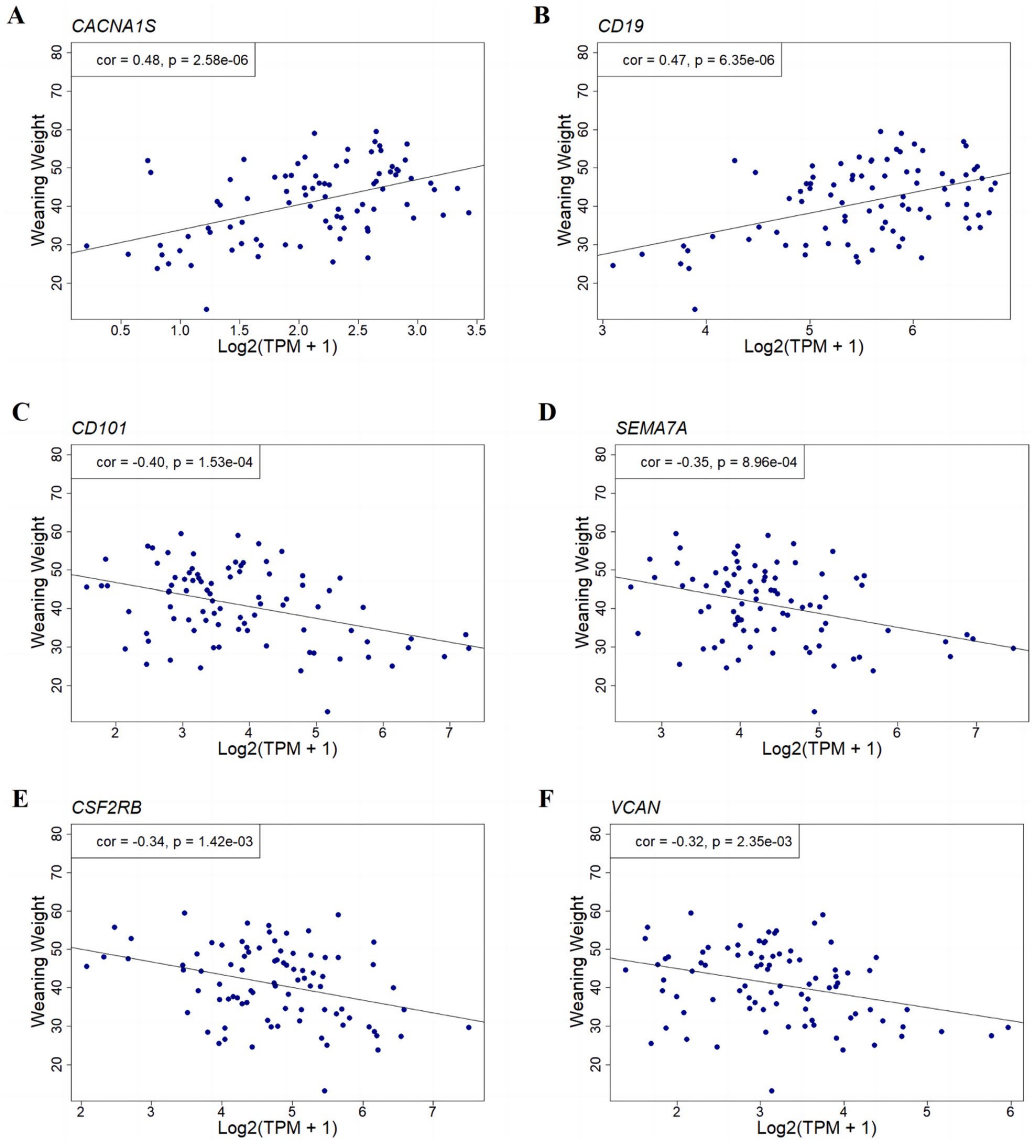
Gene examples in the gene co-expression modules associated with weaning weight. Scatter plots of expression levels of *CACNA1S*, *CD19*, *CD101*, *SEMA7A*, *CSF2RB*, and *VCAN* against weaning weight in Simmental-Holstein crossbred cattle. The x-axis represents Log2(TPM + 1) while the y-axis represents adjusted weaning weight.

The expression levels of *CACNA1S*, *SEMA7A*, *VCAN*, *CD101*, *CD19* and *CSF2RB* genes were correlated with weaning weight. *CACNA1S* (*r* = 0.48; *P* = 2.58E-06) and *CD19* (*r* = 0.47; *P* = 6.35E-06) showed positive correlation with weaning weight (Fig. 5 and Table S5). The other four genes showed a negative correlation with weaning weight, with *CD101* exhibiting the strongest correlation (*r* = 0.40; *P* = 1.53E-04) (Fig. 5; Table S5).

## Discussion

Gene expression varies across different developmental stages or physiological conditions of individuals, and exploring gene expression underlying different traits can help uncover the genetic mechanisms behind these phenotypes. Given the correlation between blood and weaning weight,^29^ we utilized blood samples for RNA-seq. A relatively large sample size (86 calves) ensured the reliability of the gene expression analyses. In this study, 38 candidate genes were identified by combining DEG analysis and WGCNA on the RNA-seq data. To understand the functions of these candidate genes, further analyses were conducted using the cattle TWAS database and the human PheWAS database. The cattle genotype-tissue expression (GTEx) atlas represents a comprehensive reference for cattle transcriptomics to date.^30^^,^^31^ By exploring the 38 candidate genes using GTEx database, this study identified *SEMA7A*, *VCAN*, *CD101*, *CD19*, *CACNA1S* and *CSF2RB* as key genes significantly associated with traits related to cattle growth and development. Furthermore, given the evolutionary conservation of genes across species^32^ and the high similarity in transcriptome profiles between cattle and humans,^21^ the association of human ortholog genes with human traits can reflect their relevance to corresponding traits in cattle. The human PheWAS database (https://atlas.ctglab.nl) is a database based on 295 GWAS studies, containing 4,155 GWAS results for 2,965 human traits.^33^ This study utilized the database to validate that the six candidate genes of this study were associated with human growth and development or immune-related traits in human. Notably, *CACNA1S* shows a significant correlation with height, white blood cells, and lymphocyte count, suggesting its key roles in growth and immune-related processes in cattle as well.

*CACNA1S* encodes the α-1s subunit of the dihydropyridine receptor (DHPR), a voltage-gated calcium channel and voltage sensor for calcium release in skeletal muscle.^34^ Mutations in *CACNA1S* were associated with susceptibility to malignant hyperthermia induced by volatile anesthetics,^35^ hypokalemic periodic paralysis,^36^^,^^37^ and thyrotoxic periodic paralysis.^38^ A previous study has shown the critical role of *CACNA1S* in muscle growth and development in yaks.^39^ *CACNA1S* was upregulated in reindeer skeletal muscle during fasting to adapt to food scarcity.^40^ Polymorphisms of *CACNA1S* were associated with carcass and meat quality traits in pigs.^41^^,^^42^ In addition, *CACNA1S* was identified as a candidate gene for several economically important traits in livestock by GWAS, such as adult weight in Santa Ines sheep,^43^ milk somatic cell counts in dairy cattle,^44^ and meat color and susceptibility to malignant hyperthermia in pigs.^45^^,^^46^ Notably, a recent study identified a missense mutation in *CACNA1S* at position 79,613,592 bp on chromosome 16 (ARS-UCD1.2) associated with muscle weakness (MW) in Holstein calves, a recessive condition affecting the standing ability of calves,^47^^,^^48^ highlighting the vital role of *CACNA1S* in the growth and development of cattle.

Growth and development of individuals are correlated with the immune system and with abnormalities often affecting normal development, including weight gain.^49^ A number of the candidate genes identified in this study were enriched in immune-related biological processes. For example, *BCL6*, *ADGRG3*, *CD19*, *CD79A*, *CD79B*, *FCRL1*, *FOSL2*, *IRS2*, *MS4A1* and *TNFRSF13C* were enriched in processes such as B cell proliferation, differentiation, and activation, while *C5AR1*, *CXCR2*, *DYSF*, *PTAFR*, *CD101* and *SLC11A1* were involved in myeloid leukocyte differentiation and activation. These genes might influence calf weaning weight by participating in the immune processes. Furthermore, based on PheWAS analysis results, the human ortholog of *CD19* showed significant association with human height and bust size, indicating that *CD19* may also play a role in both immunity and growth in cattle.

*CD19* encodes the CD19 antigen, a transmembrane glycoprotein belonging to the immunoglobulin (Ig) superfamily.^50^ CD19 plays a key role in establishing intrinsic B cell signaling thresholds via regulating both B cell receptor (BCR) dependent and independent signal transduction,^51^^,^^52^ essential for a proper immune response and widely recognized as a B cell marker. The importance of *CD19* in immunity has been extensively confirmed in both human and animal studies. However, research on its correlation with body weight and stature is mostly limited to humans. For instance, a meta-analysis of CNV associations with BMI in 191,161 European adults found a significant correlation with *CD19*.^53^

PPI analysis helps elucidate cellular signaling pathways, metabolic pathways, and disease mechanisms. The STRING database is a widely used tool for assessing and integrating protein-protein interactions.^54^ In this study, due to the positive correlation between five body size traits and weaning weight, PPI analysis was conducted on the hub genes in the central module Magenta, significantly associated with all 5 traits, using the STRING online tool. *MX1* was found to have the highest connectivity in the network, suggesting its role as a candidate gene for weaning weight in S × H cattle. *MX1*, an interferon-induced gene, encodes the MX1 protein, a GTPase playing a crucial role in defending mammalian cells against influenza A and other viruses.^55^^,^^56^ Additionally, polymorphisms in the *MX1* have been significantly associated with slaughter rate and lean meat percentage in pigs^57^ and residual feed intake in Hereford, Angus, and Simmental cattle.^58^

## Conclusion

This study employed RNA-seq technology, combined with DEG analysis and WGCNA, to conduct a bioinformatics analysis of genes related to weaning weight in S × H cattle. In total, 38 candidate genes were identified. Further analysis of these candidate genes through cattle TWAS and human ortholog PheWAS revealed *SEMA7A*, *VCAN*, *CD101*, *CD19*, *CACNA1S* and *CSF2RB* significantly associated with traits related to growth and immunity. This study provides a basis for further exploration of the genetic mechanisms underlying weaning weight in Simmental-Holstein crossbred cattle. Future exploration of the genetic variation within these six genes may further aid in the development of more precise and effective breeding strategies to achieve selective goals.

## Data Availability

The RNA-seq raw data of this study were deposited in the Sequence Read Archive (SRA) under BioProject ID PRJNA1113726.
